# *In situ* Adipogenesis in Biomaterials Without Cell Seeds: Current Status and Perspectives

**DOI:** 10.3389/fcell.2021.647149

**Published:** 2021-03-08

**Authors:** Jiqiao Yang, Chen Zhou, Jingyang Fu, Qianru Yang, Tao He, Qiuwen Tan, Qing Lv

**Affiliations:** ^1^Department of Breast Surgery, West China Hospital, Sichuan University, Chengdu, China; ^2^Laboratory of Tumor Targeted and Immune Therapy, Clinical Research Center for Breast Disease, State Key Laboratory of Biotherapy, West China Hospital, Sichuan University, Chengdu, China; ^3^West China School of Medicine/West China Hospital, Sichuan University, Chengdu, China; ^4^Laboratory of Stem Cell and Tissue Engineering, State Key Laboratory of Biotherapy, West China Hospital, Sichuan University, Chengdu, China

**Keywords:** adipose tissue engineering, biomaterials, extracellular matrix, scaffold, cell-free

## Abstract

For cosmetic and reconstructive purposes in the setting of small-volume adipose tissue damage due to aging, traumatic defects, oncological resections, and degenerative diseases, the current strategies for soft tissue replacement involve autologous fat grafts and tissue fillers with synthetic, bioactive, or tissue-engineered materials. However, they all have drawbacks such as volume shrinkage and foreign-body responses. Aiming to regenerate bioactive vascularized adipose tissue on biomaterial scaffolds, adipose tissue engineering (ATE) has emerged as a suitable substitute for soft tissue repair. The essential components of ATE include scaffolds as support, cells as raw materials for fat formation, and a tolerant local environment to allow regeneration to occur. The commonly loaded seeding cells are adipose-derived stem cells (ASCs), which are expected to induce stable and predictable adipose tissue formation. However, defects in stem cell enrichment, such as donor-site sacrifice, limit their wide application. As a promising alternative approach, cell-free bioactive scaffolds recruit endogenous cells for adipogenesis. In biomaterials without cell seeds, the key to sufficient adipogenesis relies on the recruitment of endogenous host cells and continuous induction of cell homing to scaffolds. Regeneration, rather than repair, is the fundamental dominance of an optimal mature product. To induce *in situ* adipogenesis, many researchers have focused on the mechanical and biochemical properties of scaffolds. In addition, efforts to regulate an angiogenic and adipogenic microenvironment in cell-free settings involve integrating growth factors or extracellular matrix (ECM) proteins onto bioactive scaffolds. Despite the theoretical feasibility and encouraging results in animal models, few of the reported cell-free biomaterials have been tested in humans, and failures of decellularized adipose tissues in adipogenesis have also been reported. In these cases, the most likely reason was the lack of supporting vasculature. This review summarizes the current status of biomaterials without cell seeds. Related mechanisms and influencing factors of *in situ* adipogenesis in cell-free biomaterials, dilemma in the development of biomaterials, and future perspectives are also addressed.

## Introduction

For cosmetic and reconstructive applications in the settings of adipose tissue damage due to aging and congenital or pathological conditions, such as traumatic defects, oncological resections, and degenerative diseases (Khan et al., 2012; Philips et al., 2014), the current strategies for delicate soft tissue replacement include autologous fat grafts, tissue fillers with synthetic materials, and adipose tissue engineering (ATE) (Gomillion and Burg, 2006; Choi et al., 2010). Autologous fat grafts have been acknowledged as comparatively ideal fillers because of their biocompatibility and lack of rejection immunoreaction. However, these drawbacks are also prominent. After autologous fat grafting, delayed revascularization, subsequent necrosis, and occupation of grafts by the host lead to oil cyst formation and progressive volume resorption up to 90% over time (Gomillion and Burg, 2006; Tanzi and Farè, 2009). Moreover, transplanted fat cells rarely proliferate. As a result, it is unreliable to obtain and maintain sufficient tissue augmentation only through free fat grafts. Tissue fillers remit the sacrifice of donor sites, but they often result in infection, allergic reactions, or foreign-body responses (Friedman, 1994; Gomillion and Burg, 2006; Sano et al., 2014). Similar to autologous fat grafts, they only temporarily augment volume without active self-regeneration of missing adipose tissue despite improvements in operative techniques and its popularity in the plastic market (Saylan, 2003; Itoi et al., 2010).

Aiming to regenerate bioactive vascularized adipose tissue on biomaterial scaffolds, ATE strategies have emerged as a suitable substitute for soft tissue repair (Tanzi and Farè, 2009). As outlined by Patrick (2000, 2001)) the three essential components of ATE include scaffolds as support, cells as raw materials for fat formation, and a tolerant local environment to allow these to happen. Adipogenesis can occur either *in situ* or *de novo* (Ahmed et al., 2008). *In situ* adipogenesis relies on pre-existing preadipocytes in the body, either from the surroundings or from other origins. The main techniques include fabricating and delivering biomaterials with or without bioactive factors and modifying a suitable microenvironment for the migration, proliferation, and differentiation of innate cells. This type of adipogenesis does not involve exogenous transplantation of cell seeds. *De novo* adipogenesis is based on encapsulating and transplanting cell seeds with the potential to proliferate and differentiate into adipose tissue and subsequent partial restoration of tissue functions at the expected body site (Hiraoka et al., 2006). Although preliminary research has revealed the clinical value of cell seeds, defects of stem cell enrichment, such as donor-site sacrifice, and most importantly, standardization, regulation, and concerns about biosafety, limit its wide application (Müller et al., 2020). As a promising alternative approach, cell-free bioactive scaffolds recruit endogenous cells for adipogenesis. This review summarizes the current status of biomaterials without cell seeds.

## Current Strategies and Clinical Applications of Soft Tissue Reconstruction

In clinical practice, soft tissue reconstruction can be used for cosmetic purposes and tissue defect repair. Common defect fillers include prostheses, autologous fillings, and bioactive materials. In addition, emerging tissue engineering techniques assist the above materials to improve the efficacy of reconstruction. The advantages and disadvantages of the major strategies used for the reconstruction of soft tissue defects are listed in Table 1.

**TABLE 1:** Advantages and disadvantages of major strategies for soft tissue reconstruction.

Strategies		Advantages	Disadvantages
Commercial implants		• Safe, low-complication morbidity (Duteille et al., 2018) • Mature technology	• Xenobiotic immune reaction • Capsular contracture (Prasad et al., 2019) • Migration and extrusion (Coroneos et al., 2019)
Autologous fillings	Fat grafting	• Economical and accessible (Kling et al., 2013) • Without risk of allergic reaction (Kling et al., 2013) • Share the function of both lipofilling and regeneration (Simonacci et al., 2017)	• Graft absorption and retention (Strong et al., 2015) • Unpredictable survival and durability (Strong et al., 2015)
	Flap implantation	• Greater size of the transferred tissue (Mohan et al., 2016; Abraham and Saint-Cyr, 2017) • Adequate blood supplement and low possibility of weight loss (Abraham and Saint-Cyr, 2017)	• Requirement of adequate tissue of donor site (Mohan et al., 2016; Abraham and Saint-Cyr, 2017) • Large scars (Mohan et al., 2016; Abraham and Saint-Cyr, 2017) • Time-consuming, expensive, and high requirement for surgeons and the healthcare system (Alderman et al., 2011; Panchal and Matros, 2017)
Bioactive materials		• Easy procedure • Absorbable and less rejection reaction (Lemperle et al., 2003)	• High expenses • Not used in large-volume reconstruction (Lemperle et al., 2003) • Induction of adverse reaction such as inflammation (Ferneini and Ferneini, 2016) • The need for reinjection (Ferneini and Ferneini, 2016)
Tissue engineering	Cell-assisted lipotransfer	• Improved survival rate and reduced postoperative retention (Laloze et al., 2018)	• The application is limited to small-volume fat grafting (Laloze et al., 2018)
	Acellular dermal matrix	• Overcome the problem of capsular contracture and complications (Hunsicker et al., 2017) • Providing an opportunity of immediate breast reconstruction (Salzberg, 2012; Hadad et al., 2015)	• High expenses (Glasberg, 2017)

### Prosthesis

Breast reconstruction is the most common strategy for soft tissue reconstruction for both esthetic and pathological repair purposes. Implants, including saline and silicone gel, have the advantages of low complication morbidity, mature technology, and the possibility of immediate reconstruction (Chang and Hammond, 2018). However, commercial implants may result in complications, such as infections (2.9%), capsular contracture (15–45%), and rupture (2–3.8%), and very low morbidity of implant-associated cancer, which could lead to implant loss (Prasad et al., 2019). This is due to the attachment and colonization of microorganisms and the formation of biofilms. However, the implants are also likely to suffer from migration and extrusion a long time after the procedure, which could cause patients to undergo a secondary surgery. More importantly, implants may only deal with soft tissue defects of certain shapes with available commercial products. The repair of irregular soft tissue defects or fine reconstruction of small-volume defects is less likely to be resolved by the prosthesis.

### Autologous Fillings

Autologous fillings are also widely used in soft tissue reconstruction, including autologous fat grafting and flaps. Autologous fat is considered an ideal filler because it is economical, accessible, and free of allergic reactions. It is used not only as a lipofiller but also for inducing adipose stem cells in tissue regenerative repair (Kling et al., 2013). As a result, it is widely used in facial, breast, and limb reconstruction (Simonacci et al., 2017). Moreover, the oncologic safety was also confirmed in a mouse residual breast cancer model (Silva et al., 2019). Nevertheless, the Achilles heel of fat grafting is necrosis, tissue resorption, and graft retention, with a high rate of weight and volume loss of less than 1 year after transplantation. Flap transplantation seems a better choice as it takes advantage of the vasculature of flaps and the recipient sites, which could guarantee long-term nutrition for the flaps (Abraham and Saint-Cyr, 2017). Despite the strengths of this technique, the decision regarding the size and location of the flap can only be roughly made, and the procedure always leaves obvious scars (Mohan et al., 2016; Abraham and Saint-Cyr, 2017). More importantly, the requirements of surgeons and the healthcare system limit its application (Alderman et al., 2011; Panchal and Matros, 2017).

### Bioactive Materials

Bioactive materials may also serve as soft tissue fillers, and some have already been approved by the Food and Drug Administration (FDA) and are commercially available. These materials, including hyaluronic acid, poly-L-lactic acid, calcium hydroxylapatite, and collagens, are absorbable and generally used in esthetic surgeries for small defects such as those of limbs and faces (Cohen et al., 2006; Ferneini and Ferneini, 2016). The surgical procedures using these soft tissue fillers are simple and rarely result in rejection reactions, although there are still risks of inflammation (Ferneini and Ferneini, 2016). However, these bioactive materials cannot be used to fill large volumes, such as the reconstruction of breasts (Lemperle et al., 2003). In addition, these fillers are gradually absorbed and cannot achieve long-term soft tissue reconstruction, which requires repeated injections (Ferneini and Ferneini, 2016).

### Tissue Engineering

Tissue engineering is an emerging technique for soft tissue reconstruction after the regeneration of adipose-derived stem cells (ASCs) in grafted fat. In current clinical applications, cell-assisted lipotransfer (CAL) and extracellular matrix (ECM)-based materials, based on tissue engineering techniques, can be used to compensate for the deficiencies of other materials.

Adipose-derived stem cells, along with autologous fat grafting, are the most used to improve the survival rate and reduce postoperative retention through cell enrichment. The efficiency was reported by a meta-analysis study in which 696 patients in 25 studies were reviewed and analyzed (Laloze et al., 2018). Furthermore, the advantage of CAL is only prominent in small-volume fat grafting. Kokai et al. (2019) applied an allograft adipose matrix (AAM) injection in the dorsal wrist of 15 patients, and all the patients displayed thickened wrist skin and diminished imprint of veins and tendons. However, wrist pain, injection site redness, and swelling were mostly reported in patients, and an average of 47% retention of the graft was observed.

In soft tissue regeneration, the clinical practice of the acellular dermal matrix (ADM) as a cell-free matrix dates back to as early as the 1990s. Similar to CAL, ADM is also applied with implants to overcome problems such as capsular contracture, seroma, and infection (Hunsicker et al., 2017). The matrix extract, as a soft tissue covering and prosthesis-based structural support, is sutured to the surface of the muscle, and then the prosthesis is fixed between the ADM and the muscle, which also provides an opportunity for immediate soft tissue reconstruction (Salzberg, 2012; Hadad et al., 2015). However, ADM is expensive, which limits its clinical application (Glasberg, 2017).

## Roles and Limitations of Cell Seeds in ATE

Adipose-derived stem cells are commonly loaded seeding cell populations in bioscaffolds (Bauer-Kreisel et al., 2010; Choi et al., 2010; Khan et al., 2012; Philips et al., 2014). Having been well described with the capacity to induce stable and predictable adipose tissue formation, ASCs within the stromal vascular fraction of adipose tissue have been suggested as a rational strategy to engineer mature adipose tissue (Zielins et al., 2016; Brett et al., 2017). *In vitro* studies have shown that human ASCs (hASCs) are capable of self-assembly in the presence of serum, ascorbic acid, and rosiglitazone and form dense adipocyte-containing cell sheets with an organized ECM (Vallee et al., 2009). *In vivo* experiments in animal models have comprehensively demonstrated the beneficial effects of ASC-loaded scaffolds on adipogenesis (Baglio et al., 2012). For example, when a scaffold composed of decellularized human adipose tissue and methacrylated glycol chitosan was seeded with 1 × 10^6^ allogeneic rat ASCs isolated from the epididymal fat pad, the rate of scaffold degradation and inflammatory cell infiltration and angiogenesis in the scaffold regions were enhanced as compared with those for unseeded control scaffolds (Cheung et al., 2014). hASCs showed similar properties in that mature adipose tissue was formed after hASCs were subcutaneously implanted into nude mice (Tsuji et al., 2009). Another widely researched type of cell seed is stem cells derived from bone marrow (BMSCs) isolated from mature adult tissue (Pittenger et al., 1999; Brett et al., 2017). In *in vitro* studies, human BMSCs have been found to undergo adipogenic differentiation when cultured with basic fibroblast growth factor (bFGF), also known as fibroblast growth factor-2 (FGF-2) or fibroblast growth factor-beta (FGF-β), on gelatin scaffolds or polylactide-*co*-glycolide (Hong et al., 2005; Neubauer et al., 2005). In animal studies, rabbit BMSCs treated with adipogenic medium generated new adipose tissue after they were implanted in immunocompromised mice (Choi et al., 2005). Moreover, Alhadlaq et al. (2005) subcutaneously implanted poly(ethylene glycol) dacrylate-scaffold-encapsulated human BMSCs into SCID mice after *in vitro* adipogenic differentiation and observed *de novo* lipid-containing tissue. However, although mesenchymal stem cells (MSCs) have revealed potential in ATE in animal models, results from clinical studies have scarcely been reported. Apart from serving as a reservoir for the generation of new adipocytes, cell-rich fillers also reduce wound contraction and advance wound healing, in addition to promoting adipogenesis (Debels et al., 2017).

While the value of cell seeds in adipogenesis has been well described, the shortcomings of cells are intractable. First, since seeded cells are preferably homologous, donor-site injury is unavoidable. It could be troublesome for patients with low body weight where donor-site local liposuction is unwanted (Badylak, 2002; Gomillion and Burg, 2006). In this circumstance, multiple surgical procedures consume more time before the final cosmetic results are obtained, and patients need to bear an additional financial burden. Second, the isolation and culture techniques of ASCs have not been standardized, while ASCs are very sensitive to the manufacturing processes and might be profoundly influenced, thus wrecking the predictability of cell-seeded bioactive scaffolds (Bourin et al., 2013). Moreover, the high manufacturing cost of stem cell processes has limited its application in clinical use (Lee et al., 2014). Most crucially, difficulties in cell fate control are yet to be overcome in stem cell-assisted biomaterials (Baglio et al., 2012). Especially for patients with soft tissue defects resulting from malignant tumor resection, oncological safety could be the priority when considering accepting reconstruction. However, no study has achieved long-term observation of cell culture systems for a definite proof of safety or risks for *in vivo* applications to date (Lee et al., 2014).

Furthermore, the enhancement of adipogenesis through ASCs is believed to be contemporary. CAL, that is, ASC-enriched lipotransfer, has been promoted to address the variability in fat graft retention in autologous fat grafting (Kølle et al., 2013). In autologous fat grafting, adipocytes are predisposed to apoptosis and cell death due to ischemic conditions. However, with ASC assistance, a process of dynamic remodeling of adipose tissue after non-vascularized grafting was suggested (Eto et al., 2012). Fu et al. (2013) isolated green fluorescence protein (GFP)-positive ASCs from C57BL/6J-GFP mice, mixed them with minced inguinal adipose tissue harvested from wild-type C57BL/6J mice, and then co-implanted them into BALB/c nude mice. GFP-labeled adipocytes were observed 7 days after implantation. However, the fluorescence signal intensity fell drastically within the first 14 days and continued to drop thereafter (Fu et al., 2013), suggesting insufficient durability of these new adipocytes.

## Current Applications of Biomaterials Without Cell Seeds

As a promising alternative approach, cell-free bioactive scaffolds overcome the limitations of stem cell seeding and recruit endogenous cells for adipogenesis (Hiraoka et al., 2006; Ju et al., 2014; Lih et al., 2016). The rationality and feasibility of *in situ* adipogenesis without cell seeds have been discussed (Lee et al., 2008).

Previous research has indicated that neovascularization is exclusively derived from endogenous cells through vascular remodeling, instead of exogenous cells in tissue-engineered vascular grafts (Marra et al., 2008). Rather than directly differentiating into blood vessels, exogenous cells of tissue-engineered grafts promoted the regeneration of endogenous cells (Hibino et al., 2011). Theoretically, soft tissue substitutions without seeding of exogenous cells could induce *in situ* adipogenesis and would be adequate for defect repair (Neels et al., 2004; Kelly et al., 2006; Marra et al., 2008). To induce *in situ* adipogenesis, many researchers have focused on the mechanical and biochemical properties of the adopted scaffolds. In addition, efforts have been made to regulate an angiogenic and adipogenic microenvironment in cell-free settings. The main strategies involved integrating growth factors or ECM proteins onto bioactive scaffolds (Yuksel et al., 2000a, b; Neels et al., 2004; Nillesen et al., 2007).

### Extracellular Matrices

Extracellular matrices, a microenvironment with membrane proteins and growth factors, are closely associated with the induction of cell proliferation, migration, and differentiation. In natural processes, ECMs provide an environment that facilitates cell migration and nutrient diffusion. Based on the characteristics of ECMs, acellular adipose-derived matrix is the most common seed-free scaffold used *in vivo*, as it could provide a more biomimetic environment for adipogenesis and vascularization by promoting cell adhesion and proliferation, forming neo-formative adipose tissue similar to the natural morphology of adipocytes in native adipose tissue (Wu et al., 2012) and ensuring the long-term viability of adipose stem cells inside (Rossi et al., 2018).

#### Physical Property of ECMs

Extracellular matrices are loose, porous structures composed of architectural and biochemical cues. The ECM derived from adipose tissue appears to be a white soft tissue and is always prepared with other compounds to form a hydrogel that can provide a stable cell niche and mechanical transduction pathway for adipose stem cell migration, proliferation, and differentiation (Kim et al., 2017). Flynn (2010) described the architecture of decellularized adipose tissue, which is composed of collagen fibers, decellularized vascular structures, and void spaces. The main components of the derived ECM structure include collagens, glycosaminoglycan elastin, and laminin. The structure determines the basic physical properties and basic functions of ECMs (Song et al., 2018; Thomas-Porch et al., 2018). As a physiological structure for cell activity, its porosity and extensive surface area play vital roles in cell penetration, adhesion, and proliferation (Song et al., 2018).

In addition, cell-free ECMs are highly biocompatible. According to Young et al. (2014), after the injection of decellularized adipose matrix hydrogels in mice, the mice showed high tolerance, presenting few adverse signs, and capsule formation was not observed.

#### Bioactive Components of ECMs

The bioactive components in ECMs are essential for the initiation and progression of adipogenesis and regulate the surrounding cell activities. The biochemical cues contain a widely distributed basement membrane of ECMs, including laminin, elastin, fibronectin, and glycosaminoglycans (GAGs), which promote the structural adhesion and integrity of cells, as well as endogenous growth factors, including bFGF, vascular endothelial growth factor (VEGF), hepatocyte growth factor (HGF), platelet-derived growth factor (PDGF), insulin-like growth factor 1 (IGF-1), and transforming growth factor-beta 1(TGF-β1) (Flynn, 2010; Lu et al., 2014; Kim et al., 2015; Rossi et al., 2018).

#### Mechanism of ECMs Inducing *in vivo* Adipogenesis

**FIGURE 1:**
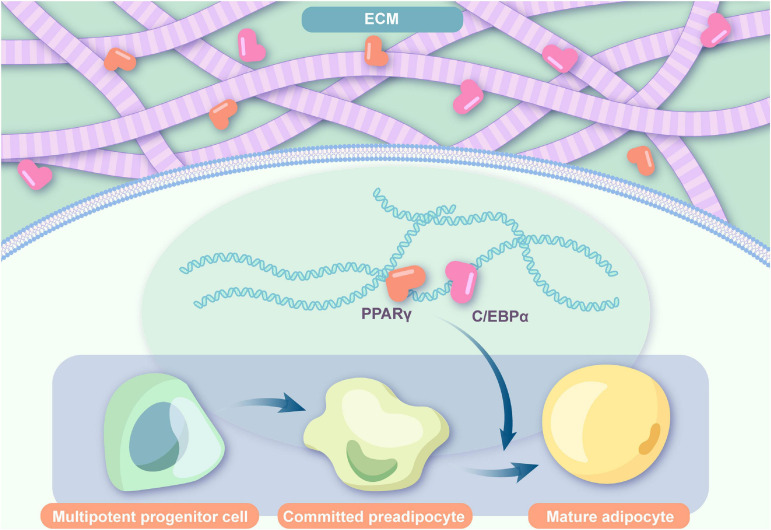
Adipogenic flow in ECMs. ECMs express major transcription factors in the adipogenic differentiation including C/EBPα and PPAR-γ and induce endogenous cell migration, proliferation, and differentiation (ECM: extracellular matrix; PPARγ: peroxisome proliferator-activated receptor gamma; C/EBPα: CCAAT/enhancer-binding protein α).

The regulation of cell recruitment, preadipocyte induction, and cell proliferation and differentiation depends on the bioactive cues inside the ECM. An effective ability to promote angiogenesis, adipo-induction, and lipid accumulation has been detected in decellularized ECM scaffolds (Jeon et al., 2020). Regardless of the addition of ASCs, the newly formed adipocytes were all host derived, suggesting that the ECM itself can recruit preadipocytes from hosts (Young et al., 2014). As observed by Kim et al. (2017), migration of host cells to the ECM scaffold was observed after angiogenesis, indicating the adipo-inducing function of vascularization factors. And this was confirmed by a study on DAT, which suggested that an enhanced concentration of bFGF could significantly improve adipogenesis (Lu et al., 2014). As one of the basic components of adipose-derived ECMs, bFGF not only promotes early angiogenesis by mobilizing endothelial cell infiltration for vascular network construction but also serves as an adipogenic-stimulating factor by inducing surrounding preadipocytes by enhancing the activity of factors that regulate both enhancer-binding protein-alpha (C/EBP_α_) and peroxisome proliferator-activated receptor-γ (PPAR-γ) expressions (Prusty et al., 2002; Marquez et al., 2017). Simultaneously, they compared the gene expression in endogenous adipose tissue with that in newly formed adipose tissue, and in addition to natural gene expression of PPAR-γ, adipocyte protein-2 (AP-2), pre-adipocyte factor-1 (Pref-1), and tumor necrosis factor α (TNF-α), the levels of C/EBP α, adiponectin, and glucose transporter-4 (Glut-4) were detected. ECMs can induce endogenous cell migration, proliferation, and differentiation by relying on a sequential multistep process that activates several transcription factors. In gene analysis, ECMs express major transcription factors in adipogenic differentiation including the expression of C/EBP_α_, which is able to induce adipogenesis in precursor cell lines that are susceptible to the expression of PPAR-γ, critical for adipogenesis promotion, as well as marker genes of the terminal process in adipogenic differentiation (Sarjeant and Stephens, 2012; Rossi et al., 2018; Hoefner et al., 2020; Figure 1). In addition, other adipogenic genes, such as aP2, adiponectin, leptin, and lipoprotein lipase, were observed in ECMs (Cheung et al., 2014; Jeon et al., 2020). These adipogenic genes may help promote a quicker induction of adipogenesis (Hoefner et al., 2020). Moreover, the increase in glycerol-3-phosphate dehydrogenase (GPDH) enzyme activity, representing the adipogenesis process, is closely associated with the adipogenic differentiation potential of ECMs without exogenous adipogenic factors (Cheung et al., 2014; Jeon et al., 2020).

#### Extracellular Matrices as Cell-Free Scaffolds

With better biocompatibility and biodegradability compared with synthetic polymers, ECMs derived from living tissues provide a suitable microenvironment that interacts with cellular components and integrates with the surrounding tissues of recipient sites (Choi et al., 2014; Sano et al., 2014). As a natural bioscaffold, the microenvironment created by ECMs is tissue specific (Choi et al., 2012; Gattazzo et al., 2014). In particular, adipose-derived ECMs naturally consist of collagen, elastin, adhesion peptides, and sophisticated substances released by adipose tissue that facilitate cell recruitment, attachment, proliferation, and differentiation (Ramirez and Rifkin, 2003). Thus, both the adipo-inductive and adipo-conductive properties are preserved (Yu et al., 2013; Han et al., 2015). The current mainstream manufacturing technique for extracting ECMs from adipose tissues is decellularization (Tanzi and Farè, 2009; Choi et al., 2010). Ideally, decellularization eliminates cells and lipids in fat, while fully preserving the three-dimensional (3D) structural and adipose-like properties of ECMs (Tanzi and Farè, 2009). However, altered decellularization methods can result in varied structural and proteomic characteristics, and all current decellularization methods inevitably result in the disruption of the ECM structure and composition to some extent. Comparison among the three decellularization measures, including enzymatic-based, detergent-based, and solvent-based methods, showed varied and inadequate decellularization and breakage of tissue architecture in the solvent agent (Thomas-Porch et al., 2018), although the composition of the functional components was similar among the three different scaffolds. It should also be noted that none of the current decellularization strategy ensures the entire removal of cell and antigen components. After decellularization, the remaining adipose matrix has pore sizes ranging from 50 to 150 μm, which is optimum for cells to migrate and settle (Yannas et al., 1989; Roehm et al., 2016). Furthermore, ECMs can be made into injectable hydrogels to fill irregularly shaped defects (Young et al., 2011; Turner et al., 2012; Giatsidis et al., 2019). At present, both porcine and human adipose tissue-derived ECMs have been reported as cell-free scaffolds for adipogenesis.

Kim et al. (2015, 2017) manufactured a soluble ECM from human adipose tissue and prepared hydrogels by mixing it with a methylcellulose solution (Choi et al., 2011). After subcutaneous injection into the backs of nude mice, accumulated intracellular lipid droplets emerged, and their levels increased significantly from weeks 2 to 3, and well-organized lobule-like structures of adipose tissue and microvessel formation were observed at week 3 (Kim et al., 2017). As tested by RT-PCR, the expression of adipocyte protein 2 (aP2), the mouse gene for adipogenesis, as well as other adipogenic genes, PPARγ, adiponectin, and leptin were expressed. In addition, angiogenesis in such cell-free hydrogels can be strengthened by angiogenic factors, such as VEGF, by recruiting and activating endogenous endothelial cells (Kim et al., 2017). Another study implanted a human acellular adipose-derived matrix in rats. Adipogenesis was observed at the periphery of the implant, and satisfying fat integration was achieved starting at the edges of the scaffold (Wu et al., 2012). In an *in vivo* study, human decellularized adipose tissue was combined with methacrylated glycol chitosan or chondroitin sulfate forming hydrogels and injected subcutaneously into Wistar rats. By week 12, scanty adipocytes were still present in the unseeded arm (Cheung et al., 2014). According to Kokai et al. (2019), a human-derived, injectable allograft adipose matrix (AAM) extracted from cadaveric human adipose contains essential collagens and growth factors that play vital roles in adipogenesis, and the implantation of the matrix in mice could support adipose regeneration *in situ*. The AAM used in this experiment was obtained from the processed human adipose tissue of cadaveric donors, where cells were removed to minimize the immune reaction, which was also used by Giatsidis et al. (2019) to reconstruct soft tissue defects in mice. The advantages of injecting AAM to induce adipogenesis were reflected in the satisfactory quality of graft and neo-formative adipocytes, where the formation of cystic areas representing adipocyte necrosis was not observed, as well as a higher density of blood vessels. Researchers also introduced an external volume expansion (EVE) model, performed as skin preconditioning using a 1–3 cm silicone cup connected to a vacuum pump and applied to the dorsum of animals to suction at 25 mmHg six times per day. Compared to AAM alone, incorporation of both EVE and AAM resulted in better adipogenesis, presenting as a greater number of adipocytes and satisfactory angiogenesis. The application of the graft was also studied in humans, and satisfactorily enhanced skin and subcutaneous tissue was harvested with some minor observations, including wrist pain, redness, and swelling, as well as graft retention (Kokai et al., 2019). Easily available with no need for donor-site sacrifice, the acellular porcine adipose matrix has been thought to provide properties comparable to those of acellular human adipose matrices (Roehm et al., 2016). Tan et al. (2019) introduced a hydrogel from acellular porcine adipose tissue (HAPA) as a novel allogenic biomaterial that could induce adipocyte regeneration in wound tissue. Compared with those in the control group, better adipogenesis, angiogenesis, and wound healing were observed in mice injected with HAPA. Although neogenetic adipocytes and vessels in the ASC-seeded HAPA group were more satisfactory in this study than those for HAPA alone, the angiogenesis microenvironment that HAPA provided could trigger adipogenesis, probably by activating the associated transcription factors and inducing adipocyte regeneration. A novel adipogenic matrix, Adipogel, is derived from the ECMs of decellularized porcine adipose tissue and can also be manufactured from other species, including rats and humans (Debels et al., 2017). Subcutaneous thoracic implantation of Adipogel in rats depicts the clinical setting where soft tissue defects are adjacent to existing fat. Eight weeks after thoracic implantation, the majority of Adipogel implants demonstrated large clusters of adipocytes, although not as closely packed as in mature neighboring fat (Debels et al., 2017). After lumber implantations that mimicked defects where there was minimal adjacent fat, such as scarred tissue, implants were hardly found. However, adipocytes were observed wherever the implants were identified. At both implantation sites, differentiating preadipocytes were found among mature adipocytes, as depicted by perilipin staining. Thus, this cell-free biomaterial effectively maintained a space for tissue ingrowth and induced adipogenic differentiation from migrated endogenous cells (Debels et al., 2017).

### Natural or Synthetic Cell-Free Bioactive Scaffolds

Biosynthetic polymers have been widely investigated as scaffolds for ATE because their physical properties are modifiable and can be scalable. However, synthetic scaffolds can result in fibrous encapsulation or a severe inflammatory response and adverse events such as extrusion, cyst formation, and overlying skin ulceration. These inherent shortcomings have limited its development (O’Brien, 2011; Brett et al., 2017).

#### Gelatin Cryogel Scaffold

Chang et al. (2018) reported a synthetic cell-free Gelatin Cryogel (GC) scaffold coated with polydopamine (PDA) and immobilized platelets (Plts) that formed an injectable and safe chamber to regenerate large adipose tissues. This chamber sustained high levels of PDGF and VEGF released from Plts, which indicates more blood supplementation and less hypoxia. Moreover, GC is degradable, and the host cells can migrate into scaffolds after degradation and start a natural fiber network generation, which forms a natural ECM and induces less inflammatory reaction. These factors resulted in a large volume of adipose tissue.

#### Hyaluronan Hydrogel

Hyaluronan (HA) hydrogels have been widely used as defect fillers and have been used for tissue engineering in bones (Hulsart-Billström et al., 2018), cartilage (Toh et al., 2010), intervertebral disc (Calderon et al., 2010), and soft tissue (Sarkanen et al., 2012). Several injectable HA hydrogel materials have been approved by the FDA for clinical use, and HA hydrogel-related adverse events have been minimal (Rohrich et al., 2007). Sarkanen et al. (2011) incorporated adipose tissue extracts, a blend of a wide variety of components of mature adipose tissue, including VEGF, bFGF, adiponectin, angiogenin, and interleukin 6, into HA hydrogel to develop an acellular implant, namely, the ATE-HA implant. The implant gradually released bioactive factors *ex vivo*. In *in vivo* studies, the implants were placed under the dorsal subcutis of rodents, and triglyceride accumulation emerged as early as 1 week after implantation. Eventually, well-vascularized and nerved adipose tissues were observed at 40 weeks. The fat within the biomaterials was densely integrated and vessel accompanied, which was very similar to the native adipose tissue (Sarkanen et al., 2012). This acellular implant was considered to have performed well in terms of compatibility, bioactivity, and sustainability and has considerable potential for use in tissue engineering for sustained reconstruction of soft tissue defects.

#### 3D-Bioprinted Scaffold

In a study with 3D-printed biomaterials, polycaprolactone (PCL) particles were melted and 3D-printed as cylinders. Neutralized type I collagen solution was infused into the microchannels of the PCL scaffold, followed by gelation. PCL scaffolds were implanted in the inguinal fat pad *in vivo* in mice, and *in situ* adipogenesis was observed 4 weeks after implantation (Shah et al., 2017). However, such adipogenesis relied on the seeding of Pyrintegrin, an assisting substance that will be described in detail later in this review.

## Failures in *in situ* Adipogenesis

Despite the theoretical feasibility and encouraging results in animal models as well as *in vitro* experiments, failures of decellularized adipose tissues in adipogenesis have also been reported (Friedman, 1994; Yu et al., 2013). For example, an ECM product, Matrigel, was adipogenic only when it is in direct contact with adjacent fat in a mouse model (Kelly et al., 2006). The researchers inferred that the autograft could provide ECM components, paracrine factors, or chemokines that promote adipogenesis. For the chamber without cell seeding, the lack of a microenvironment for cell migration, adhesion, and proliferation was the probable reason for the failure. In an *in vivo* study, no vacuolated fat-like cells were found in bare bovine type I collagen 3 weeks after implantation in rat dorsal muscle if unseeded. Similarly, unsatisfactory adipogenic induction from direct implantation of decellularized adipose tissues alone was also reported (Zhang et al., 2016). In these cases, the most proposed reasons were a lack of supporting vasculature because of inadequate angiogenesis factors such as bFGF. A similar result was also observed for the decellularized ECM hydrogel that lacks bFGF, which could result in the disappearance of existing adipose tissue, indicating the vital role of bFGF during adipogenesis (Lu et al., 2014). In another study, hydrogel alone could induce little adipogenesis, while the addition of ASCs and/or TG could greatly improve adipogenesis by promoting vascularization, as stimulation of adipogenesis was observed following the formation of blood vessels. As a result, angiogenesis is closely associated with adipogenesis (Young et al., 2014). In summary, adipogenesis in unseeded materials should possess the ability to provide an appropriate environment and induce enough angiogenesis.

## Factors That Affect *in situ* Adipogenesis in Cell-Free Biomaterials

In biomaterials without cell seeds, the key to sufficient adipogenesis relies on the recruitment of endogenous host cells and continuous induction of cell homing to scaffolds because no exogenous mesenchymal cells could be utilized. Regeneration, rather than repair, is the fundamental dominance of an optimal mature product. In addition, it is expected to degrade smoothly and eventually be replaced by healthy functional host fat tissue. To achieve such properties, the adipogenic mechanisms in cell-free biomaterials should be explored. Influencing factors, including but not limited to recruiting potency, growth factor integration, biocompatibility, and mechanical characteristics, should be adequately considered and balanced.

### Recruitment and Origin of Host Cells

*In vivo* studies have revealed better adipogenic efficacy of adipose-derived acellular matrix at fat-rich implantation sites than at fat-bare sites (Debels et al., 2017). For quite some time, it was widely believed that the cells migrating to cell-free scaffolds mainly come from the surrounding tissues. An up-to-date research suggested that hydrogels of decellularized porcine adipose tissue induce host-derived adipogenesis in nude mice. Unlike the ASC-seeded hydrogel, where islet-like adipocyte clusters seen with adipogenesis in the basal region deteriorated over time, adipocytes were scattered in the unseeded hydrogel with more adipogenesis observed on the basal side (Tan et al., 2017). As proposed by the authors, this disparity in the patterns of adipose tissue remodeling between seeded and unseeded matrices might be associated with differences in the cell origins of adipocytes. While little fat tissue existed subcutaneously in nude mice, adipocytes that emerged in the unseeded hydrogel were less likely to come from the surrounding fat.

Nearly any tissue in the body harbors some sort of progenitor cells, including, but not limited to, fat, muscle, dermis, brain, liver, heart, placenta, and circulating blood (Gage, 2000; Liu et al., 2009; Kalinina et al., 2011). The alternative origin of precursor cells could be bone marrow other than the direct addition of preadipocytes in an invasive way. The exact sources and migration routes of host cells remain unsolved. More specifically, are there any BMSCs homing to the cell-free scaffolds? If any, what percentage of cells was involved, and how did it occur? Answers to these questions are priceless for the development of bioactive fillers because bone marrow remains hemopoietic throughout the life span of a person and could be a natural inexhaustible cell reservoir for sustainable adipogenesis. Clinically, this might lead to a very stable long-term volume maintenance at the implant site.

**FIGURE 2:**
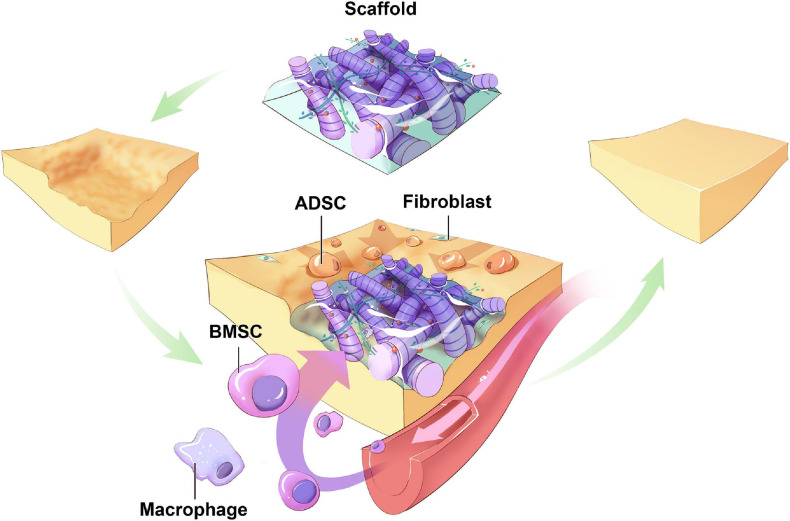
Hypothetic sources of host cells in cell-free biomaterials. Around the biomaterials implanted in the soft tissue defects, ADSCs migrate from surrounding tissues toward the scaffolds and differentiate. BMSCs mobilize from niches in bone marrow into circulation, migrate to and colonize on the scaffolds, proliferation and differentiation into mature adipocytes. Other types of progenitor cells from other body parts may also serve as origins of host cells (ADSC: adipose-derived stem cell; BMSC: bone marrow-derived mesenchymal cell).

The recruitment of BMSCs to regenerate the expected tissue is a sophisticated cascade of interactions. The remote signal is sensed before BMSCs are mobilized from niches in the bone marrow into the circulation. Then, BMSCs transit in the vascular system, adhere to the surface of endothelial cells across the vessels, and migrate toward the target areas. Subsequently, BMSCs proliferated and differentiated into mature adipocytes *in situ* (Liu et al., 2009; Figure 2).

Cytokines and chemokines as chemoattractants largely influence the mobilization and homing of progenitor cells (Agrawal et al., 2011; Sicari et al., 2014). These chemoattractants can be released from the ECM scaffolds. Stem cell-activating molecules such as VEGF, HGF, IGF-1, and PDGF promote the recruitment of host ASCs from the nearby adipose tissue (Hu et al., 2015; Stuermer et al., 2015). While stromal cell-derived factor (SDF)-1a and its receptor, chemokine receptor type 4 (CXCR4), construct the most important functioning pathway in the homing of BMSCs (Liu et al., 2009), designing cell-free biomaterials that regulate the SDF-1-CXCR4 axis has not yet been attempted. Adipocytes are not the only type of cells in fat, and other types include inflammatory cells, pericytes, and fibroblasts (Eto et al., 2009; Yoshimura et al., 2011). Even though they constitute less than 10% of the fat volume summed up, they are indispensable in the whole picture of adipose tissue remodeling, and thus, they should not be neglected in the development of bioscaffolds.

In addition, implantation of unseeded materials induces the infiltration of immune cells such as neutrophils and macrophages from hosts. Adipose tissue macrophages, especially the M2 phenotype, are closely associated with working as major immune cell types in adipose tissues that are thought to play important roles in adipose tissue remodeling, which is an essential procedure during adipogenesis (Brown et al., 2012). Lilja et al. (2013) established a chamber to induce adipogenesis and found that the first host cells that migrated were macrophages and that adipose precursor cells accounted for only a minority of the migrated cells. Furthermore, the macrophages recruited from the host to ECM hydrogels induced adipose tissue remodeling and adipogenic activities (Kim et al., 2017).

### Enrichment of Growth Factors and Cytokines

Endogenous adipokines include bFGF, VEGF, TGF-β1, placental growth factor (PGF), IGF-1, HGF, and PDGF (Kim et al., 2017). One of the most widely adopted angiogenic factors to be enriched in biometric is bFGF (Lu et al., 2014). BFGF is naturally expressed in adipose tissue, and subcutaneous and omental adipose tissue mass can be regulated by bFGF, which is locally produced by adipocytes (Gabrielsson et al., 2002; Brett et al., 2017). Zhang et al. (2016) loaded allogeneic decellularized adipose tissue with bFGF and stem cells. Twelve weeks after injection in mice, only the bFGF-loaded implants resulted in significant *in situ* formation of adipose tissue that closely resembled the inherent fat in tissue architecture, whereas all the non-loaded implants degenerated (Zhang et al., 2016). bFGF was also incorporated onto minced collagen sponges and gelatin microspheres for release control. In the rabbit model, the histological area of *in situ* adipogenesis was enhanced over time by repeated administration (Lu et al., 2014). Furthermore, bFGF induced the formation of adipose tissue accompanied by microvasculature when subcutaneously injected into BALB/c mice with gelatin microspheres and Matrigel (Tabata et al., 2000).

Several other growth factors have been used in animal models. In a study by Yuksel et al., soluble IGF-1 was bound to poly(lactic-*co*-glycolic) acid (PLGA)/PEG microspheres and injected subcutaneously into the deep muscular fascia of the abdominal muscle wall in rats. Four weeks after injection, adipocyte formation was observed within the non-adipocyte cell depots (Yuksel et al., 2000c). Furthermore, the combination of growth factors (e.g., combination of bFGF, VEGF-A, and PDGF-BB) also significantly improved the formation of adipose tissue within subcutaneously implanted chambers in immunocompromised mice (Ting et al., 2014).

The effects of the incorporated growth factors on adipogenesis are dose sensitive. In an *in vivo* study on bFGF-loaded scaffolds, *in situ* adipogenesis accompanied with angiogenesis was observed in the implanted scaffold with 1.0 μg of bFGF per defect, and the extent was less at lower and higher bFGF doses. Presumably, an inadequate dose of bFGF was insufficient to exert its angiogenic or adipogenic effect, while bFGF overdose caused an inflammatory response at the implanted site and led to accelerated infiltration of fibrous tissues into the scaffold (Hiraoka et al., 2006). Therefore, finding an optimal concentration rather than simply adopting a maximal dose is pivotal in the development of biomaterials without cell seeds (Agrawal et al., 2011).

### Supporting Role of Structural Proteins

A considerable portion of efforts in the development of biomaterials has been devoted to enriching biomaterials with structural ECM components (Badylak, 2002). In ECM biomaterials, structural proteins offer architectural support to cells. Collagen types I–VI are dominant in structural proteins of adipose-derived ECM (Divoux and Clement, 2011). Interacting with other molecules, collagen VI regulates the extension of fat and insulin sensitivity (Mariman and Wang, 2010). Other structural proteins, including elastin, fibronectin, and glycosaminoglycans, may profoundly regulate cellular behaviors during adipogenesis (Nakajima et al., 2002). When structural proteins are applied, it should be noted that the effects of incorporated structural proteins in adipogenesis are dose sensitive. Specifically, while maintaining structural integrity and facilitating *in vivo* adipogenesis, excessive deposition of collagen is associated with pathological fibrosis during fat remodeling (Chun, 2012).

### Incorporation of Other Substances

Other substances or particles have been suggested to assist adipogenesis in cell-free biomaterials. Pyrintegrin is a 2,4-disubstituted pyrimidine that induces the activation of β1 integrin and multiple growth factor receptors, including FGF receptor 1 (FGFR1), IGF-1 receptor (IGFR1), epidermal growth factor receptor 1 (EGFR1), and human epidermal growth factor receptor 2 (HER2) (Xu et al., 2010). It is capable of improving the survival of human embryonic stem cells (Xu et al., 2010), which upregulates PPARγ and C/EBPα, a pair of molecules that promote adipose cell differentiation toward fibroblasts or myoblasts (Kawai et al., 1985). Shah et al. (2017) implanted Pyrintegrin-adsorbed 3D-bioprinted PCL scaffolds in the inguinal fat pad *in vivo* in C57BL/6 mice and observed the formation of adipose tissue that was positive for Oil Red O. Moreover, mouse PPARγ was significantly overexpressed in the harvested tissue within the PCL collagen gel scaffolds (Shah et al., 2017). Given that no cell seeds were transplanted, the new adipose tissue formed by host cells in the native environment demonstrated *in situ* adipogenesis with no need for stem cell assistance.

## Conclusion and Future Perspectives

On the one hand, cell-seeded materials remain the mainstream with great potential in the field of fine soft tissue reconstruction with biomaterials. On the other hand, the inherent shortcomings of stem cells make cell-free biomaterials an important alternative option. Adipogenic biomaterials without cell seeds overcome the limitations of stem cell enrichment, including donor-site sacrifice, potential oncological concerns, troublesome manufacturing processes, and extra cost.

While bearing notable advantages, the most significant restriction of cell-free bioscaffolds lies in the recruitment of endogenous host cells. Owing to the difficulties in cell labeling, the origins and mobilizing routes of host cells in *in situ* adipogenesis remain ambiguous. Once the potential pathways of BMSC homing are sufficiently explored and steadily manipulated in implanted biomaterials, long-term clinical volume maintenance could be realistic. In the research of cell-free biomaterials, apart from the extensively attempted importation of structural proteins and growth factors, some other influencing factors have been mentioned but have yet to be thoroughly investigated, including stiffness and pore diameters of scaffold and genetic homology of ECMs.

Despite the encouraging results of *in vivo* studies, only a few of the reported cell-free biomaterials have been tested in humans. Therefore, more efforts are needed to create practical and affordable biomaterials with optimal long-term cosmetic outcomes.

## Author Contributions

All authors made substantial contributions to the conception of this review and the critical appraisal of the literature summarized herein, wrote the manuscript, and approved the final version of this article before submission.

## Conflict of Interest

The authors declare that the research was conducted in the absence of any commercial or financial relationships that could be construed as a potential conflict of interest.
